# Author response to: Comment on: Evolution of transoral endoscopic thyroidectomy vestibular approach according to the IDEAL framework

**DOI:** 10.1093/bjs/znac236

**Published:** 2022-07-27

**Authors:** Shen Han Lee, Ram Moorthy, Sidhartha Nagala

**Affiliations:** Department of Otorhinolaryngology, Hospital Sultanah Bahiyah, Kedah, Malaysia; Department of Otolaryngology—Head and Neck Surgery, Royal Berkshire Hospital, Reading, UK; Department of Otolaryngology—Head and Neck Surgery, Wexham Park Hospital, Frimley Health NHS Foundation Trust, Wexham, UK; Department of Otolaryngology—Head and Neck Surgery, Royal Berkshire Hospital, Reading, UK

*Dear Editor*

We would like to thank Alkaissy *et al.* for their interest in our paper^1^ and for discussing it in their journal club.

We chose to write a narrative review to track the development of the transoral endoscopic thyroidectomy vestibular approach (TOETVA) in order to avoid losing the historical narrative within the restrictive confines of a systematic review. As such, neither a rigid search strategy nor predefined inclusion/exclusion criteria were applied. However, keywords used to search the literature included transoral thyroidectomy, remote-access thyroidectomy, minimally invasive thyroidectomy, transoral endoscopic thyroidectomy vestibular approach, and TOETVA. In addition, original articles referenced from published review articles were also reviewed for inclusion. We acknowledge that the non-systematic manner of study selection may potentially have led to biases, as is inherent in the nature of narrative reviews.

Ideally, a non-inferiority randomized controlled trial (RCT) should be performed to compare TOETVA against open surgery. However, one should consider that the patients most likely to choose TOETVA are those who strongly desire to avoid a visible neck scar. On the one hand, it may be ethically questionable to enrol them in an RCT where there is a possibility of being randomized to the open thyroidectomy arm. On the other hand, excluding them will remove a significant proportion of patients who stand to benefit from TOETVA. To this end, the importance of informed consent in participating in such a trial cannot be overemphasized.

Finally, TOETVA may perhaps be limited in the magnitude of disease burden that it addresses and its cost-effectiveness is yet to be established. However, the authors raise a good point regarding potential benefits from better visualization using high-definition endoscopic optics. Well designed RCTs based on multi-institutional collaborations and international registry studies with systematic collection of outcomes data may reveal more unanticipated benefits of TOETVA in the future.

*Disclosure*. The authors declare no conflict of interest.
